# Supplementary material for: Identification and Characterization of Alternatively Spliced Transcript Isoforms of IRX4 in Prostate Cancer
Source: Genes (Basel). 2021 Apr 21;12(5):615. doi: 10.3390/genes12050615 (PMC8143155; doi:10.3390/genes12050615)
Supplement: Supplementary file 1 [file genes-12-00615-s001.zip › genes-1154286-supplementary.pdf]

Supplementary Material: Identification and characterization of alternatively spliced transcript isoforms of *IRX4* in Prostate Cancer.

Achala Fernando, Chamikara Liyanage, Afshin Moradi, Panchadsaram Janaththani and Jyotsna Batra

0	70	80	90	100	110	120	130	140
predicted sequence (HTR011738.5.69.3)	TTCTGCAACT'	CAGAACATCC'	CCACTTCAG-'	----------'	----------'	----------'	----------'	----------'
Variant 6	TTCTGCAACT'	CAGAACATCC'	CCACTTCAGG'	TTTGCGAGAC'	CAGGTTTTAA'	AGCAAGCGGG	AAATCAGCTC'	CTTCCTGCCG'
Variant 8	----------'	----------'	---------G	TTTGCGAGAC	CAGGTTTTAA	AGCAAGCGGG	AAATCAGCTC	CTTCCTGCCG
Variant 10	----------'	----------'	----------'	----------'	----------'	----------'	----------'	----------'
Variant 12	----------'	----------'	----------'	----------'	----------'	----------'	----------'	----------'
	EXON 3a							

	150	160	170	180	190	200	210	220
predicted sequence (HTR011738.5.69.3)	----------'	----------'	----------'	----------'	----------'	----------'	----------'	----------'
Variant 6	GGGCGTAGGG	CTGCCCAGGA'	ATCCTTGCCA'	AGCCTTTGGG'	AGTGGGGGGC	TCCTAGGAGA'	GAGGCTGGAG	TGGGGTTGCC'
Variant 8	GGGCGTAGGG	CTGCCCAGGA	ATCCTTGCCA	AGCCTTTGGG	AGTGGGGGGC	TCCTAGGAGA	GAGGCTGGAG	TGGGGTTGCC
Variant 10	----------'	----------'	----------'	----------'	----------'	----------'	----------'	----------'
Variant 12	----------'	----------'	----------'	----------'	----------'	----------'	----------'	----------'
						INTRON 4		

	230	240	250	260	270	280	290	300
predicted sequence (HTR011738.5.69.3)	----------'	----------'	----------'	---------A'	ACAGCTTTGA'	TTCCAAGGAT'	GGTTCGGGAT'	CTGCGCATGG'
Variant 6	TCCCTGGAAA'	CTGCGACCCC'	AGACTCTTGG'	CTCCCACAGA'	ACAGCTTTGA'	TTCCAAGGAT'	GGTTCGGGAT'	CTGCGCATGG'
Variant 8	TCCCTGGAAA	CTGCGACCCC	AGACTCTTGG	CTCCCACAGA	ACAGCTTTGA	TTCCAAGGAT	GGTTCGGGAT	CTGCGCATGG
Variant 10	----------'	----------'	----------'	---------A	ACAGCTTTGA	TTCCAAGGAT	GGTTCGGGAT	CTGCGCATGG
Variant 12	----------'	----------'	----------'	---------A	ACAGCTTTGA	TTCCAAGGAT	GGTTCGGGAT	CTGCGCATGG

	310	320	330	340	350	360	370	380
predicted sequence (HTR011738.5.69.3)	GGGCCTGGCA'	CCAGCCGCTG'	CCGCCTACTA'	CCCTTACGAG'	CCAGCTCTGG'	GCCAGTACCC'	CTATGACAG-'	----------'
Variant 6	GGGCCTGGCA'	CCAGCCGCTG'	CCGCCTACTA'	CCCTTACGAG'	CCAGCTCTGG'	GCCAGTACCC'	CTATGACAG-'	----------'
Variant 8	GGGCCTGGCA	CCAGCCGCTG	CCGCCTACTA	CCCTTACGAG	CCAGCTCTGG	GCCAGTACCC	CTATGACAG-'	----------'
Variant 10	GGGCCTGGCA	CCAGCCGCTG	CCGCCTACTA	CCCTTACGAG	CCAGCTCTGG	GCCAGTACCC	CTATGACAG-'	----------'
Variant 12	GGGCCTGGCA	CCAGCCGCTG	CCGCCTACTA	CCCTTACGAG	CCAGCTCTGG	GCCAGTACCC	CTATGACAGA	GGGCCCAGCC
					EXON 3			

	390	400	410	420	430	440	450	460
predicted sequence (HTR011738.5.69.3)	----------'	----------'	-------GTA'	TGGAACCATG'	GACAGCGGCA	CGCGGCGCAA'	GAACGCCACG'	CGCGAGACCA'
Variant 6	----------'	----------'	-------GTA'	TGGAACCATG'	GACAGCGGCA	CGCGGCGCAA'	GAACGCCACG'	CGCGAGACCA'
Variant 8	----------'	----------'	-------GTA	TGGAACCATG	GACAGCGGCA	CGCGGCGCAA	GAACGCCACG	CGCGAGACCA
Variant 10	----------'	----------'	----------'	----------'	----------'	----------'	----------'	----------'
Variant 12	TGAGCCCATT	GCAGAGCCTG	TCTCCAGGTA	TGGAACCATG	GACAGCGGCA	CGCGGCGCAA	GAACGCCACG	CGCGAGACCA

	470	480	490	500	510	520	530	540
predicted sequence (HTR011738.5.69.3)	CCAGCACGCT'	CAAGGCCTGG'	CTGCAGGAGC'	ACCGCAAGAA'	CCCCTACCCC'	ACCAAGGGCG'	AGAAGATCAT'	GCTGGCCATC'
Variant 6	CCAGCACGCT'	CAAGGCCTGG'	CTGCAGGAGC'	ACCGCAAGAA'	CCCCTACCCC'	ACCAAGGGCG'	AGAAGATCAT'	GCTGGCCATC'
Variant 8	CCAGCACGCT	CAAGGCCTGG	CTGCAGGAGC	ACCGCAAGAA	CCCCTACCCC	ACCAAGGGCG	AGAAGATCAT	GCTGGCCATC
Variant 10	----------'	----------'	----------'	----------'	----------'	----------'	----------'	----------'
Variant 12	CCAGCACGCT	CAAGGCCTGG	CTGCAGGAGC	ACCGCAAGAA	CCCCTACCCC	ACCAAGGGCG	AGAAGATCAT	GCTGGCCATC
					EXON 5			

	550	560	570	580	590	600		
predicted sequence (HTR011738.5.69.3)	ATCACCAAGA'	TGACCCTCAC'	ACAGGTCTCC'	ACCTGGTTCG'	CCAACGCGCG'	CCGGCGCCTC'		
Variant 6	ATCACCAAGA'	TGACCCTCAC'	ACAGGTCTCC'	ACCTGGTTCG'	CCAACGCGCG'	CCGGCGCCTC'		
Variant 8	ATCACCAAGA	TGACCCTCAC	ACAGGTCTCC	ACCTGGTTCG	CCAACGCGCG	CCGGCGCCTC		
Variant 10	----------'	----------'	------TCTCC	ACCTGGTTCG	CCAACGCGCG	CCGGCGCCTC		
Variant 12	ATCACCAAGA	TGACCCTCAC	ACAGGTCTCC	ACCTGGTTCG	CCAACGCGCG	CCGGCGCCTC		

Figure S1. Alignment of predicted *IRX4* transcripts 6, 8, 10 and 12 sequences.

Alignment of DNA sequences of predicted *IRX4* transcripts, HTR011738.5.69.3 (predicted seq for variant 6), 6 (real variant 6), 8, 10 and 12 transcript variants have shown in the figure with respect to their exon 3a, intron 4, exon 3 and exon 5. The base-pair sequences change predominantly in these regions while other coding regions similar to each other and other *IRX4* variants as we expected. The numbers denote the base pair number with respect to variant 6 and the sequences have been grouped in the quantity of 10 base pairs for easy identification. The red colour letters denote the extended exon 3 and 5 of the 5’UTR region for variant 12 and coding starts at exon 5.

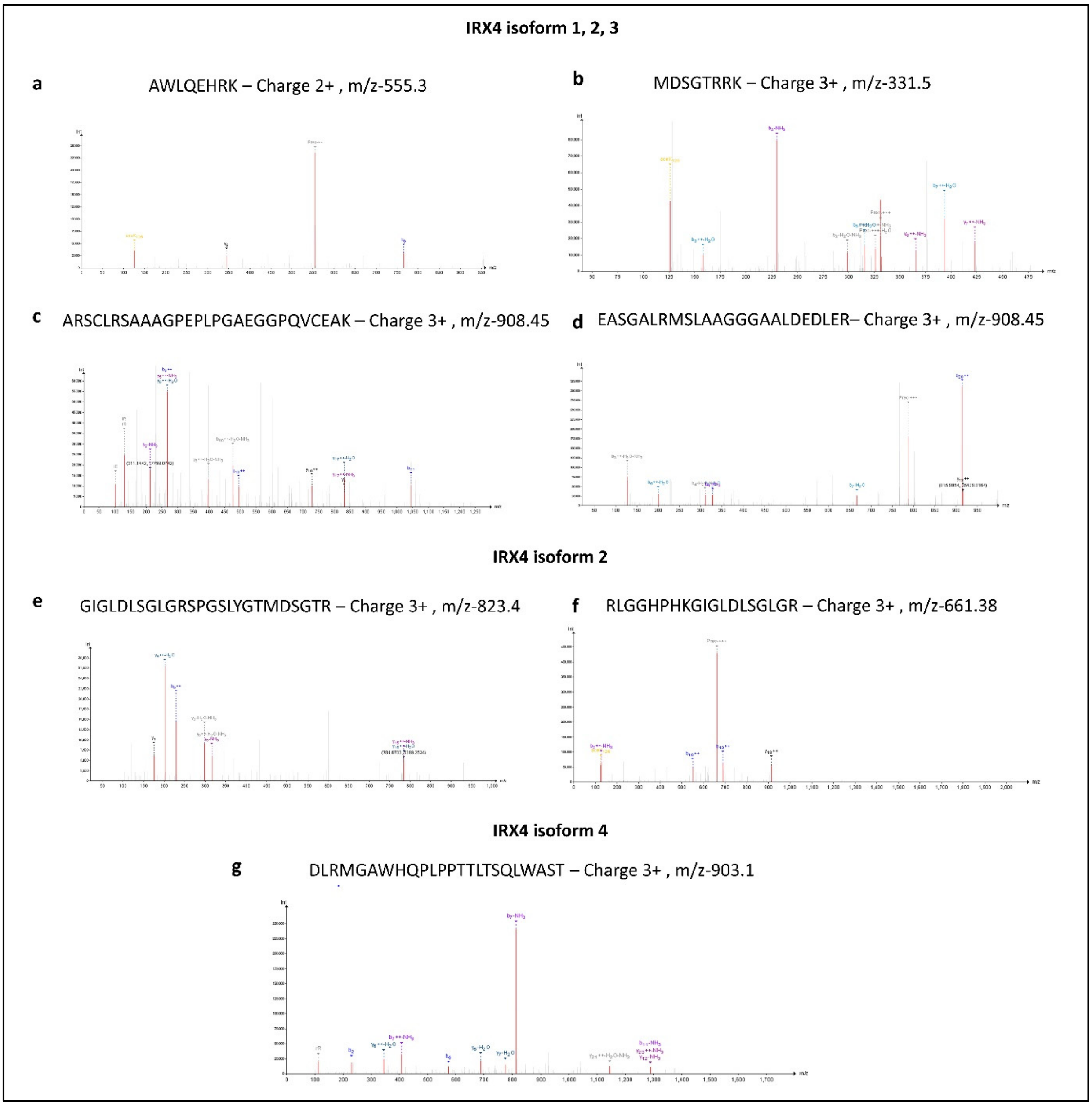

Figure S2. Identification of IRX4 isoform-specific peptides by reprocessing LNCaP LC-MS/MS data retrieved from PRIDE database. Each subfigure represents MS/MS ion fragmentation spectrum, charge, mass/charge ratio (m/z) for each peptide identified for IRX4 isoforms. a, b, c, d represent common peptides for IRX4 isoform 1, 2 and 3. e, f represents the specific peptides for IRX4 isoform 2. g represents the specific peptide for IRX4 isoform 4.

Table S1. Primer sequences for *IRX4* transcripts used in the study.

Primer set(target variants)	Forward Primer (FP)	Reverse primer (RP)	The expected PCR product(bp)	The resulted PCR product (bp)
1(1-5)	AGGGCTATGGCAACTACGTG	GCATGATCTTCTCGCCCTTG	369,291	369,291
2(6,7)	GCTCCACCACACACTGCTTCT	GCATGATCTTCTCGCCCTTG	281, 359	451, 529
3(8,9)	AGGGCTATGGCAACTACGTG	TCCAGCCTCTCTCCTAGGAGCC	265	176
4(10,11)	AGGGCTATGGCAACTACGTG	TCGTCCAAGTCACTAAGCTCC	544,622	386,464
5(12)	TGCAGAGCCTGTCTCCAGGTA	TCGTCCAAGTCACTAAGCTCC	396	396
6(6)	GCTCCACCACACACTGCTTCT	GTCCATGGTTCCATACCTGTCA	342	342
7(7)	GCTCCACCACACACTGCTTCT	GATGACCGCCTAGCCGTTTG	349	349
8(8)	TTCTACTCGCTGGTTTGCGA	GTCCATGGTTCCATACCTGTCA	308	308
9(9)	TTCTACTCGCTGGTTTGCGA	GATGACCGCCTAGCCGTTTG	315	315
10(10)	CTATGACAGTCTCCACCTGGTT	TCGTCCAAGTCACTAAGCTCC	229	229
11(11)	GCTCCCTTCTCCACCTGGTT	TCGTCCAAGTCACTAAGCTCC	227	227
RPL32	TTCATCCGGCACCAGTCA	GGCCCTTGAATCTTCTACGA	97	97
qPCR primers				
12(all)	CGCCTTCTACTCGCTGAACA	AGAGCTGGCTCGTAAGGGTA	105	105
13(1,3,5)	CGCCTTCTACTCGCTGAACA	GTCCATGGTTCCATACCTGTCA	142	142
14(2,4)	CGCCTTCTACTCGCTGAACA	TTCCTTTATGGGGATGACCGC	161	161
15(6,7)	GCTCCACCACACACTGCTTCT	TCCAGCCTCTCTCCTAGGAGCC	166	166
16(8,9)	TTCTACTCGCTGGTTTGCGA	TCCAGCCTCTCTCCTAGGAGCC	132	132
17(10)	CTATGACAGTCTCCACCTGGTT	CTGCGTTCTTGGAGCTCTT	180	180
18(11)	GCTCCCTTCTCCACCTGGTT	CTGCGTTCTTGGAGCTCTT	178	178
19(12)	TGCAGAGCCTGTCTCCAGGTA	GCATGATCTTCTCGCCCCTT	143	143
RPL32	CCCTTGTGAAGCCCAAGA	GACTGGTGCCGGATGAACTT	59	59
